# Interplay of Atrial Arrhythmia and Chronic Heart Failure: A Population-Based Analysis of Hospital Outcomes in Germany

**DOI:** 10.7150/ijms.131856

**Published:** 2026-06-25

**Authors:** Anastasia Janina Hobbach, Jannik Feld, Jürgen Reinhard Sindermann, Wolfgang Albrecht Linke, Holger Reinecke

**Affiliations:** 1 Department of Cardiology I, Coronary, Peripheral Vascular Disease and Heart Failure, University Hospital Münster, Münster, Germany.; 2 Institute of Biostatistics and Clinical Research, University of Münster, Münster, Germany.; 3 Institute of Physiology II, University of Münster, 48149, Münster, Germany.

**Keywords:** chronic heart failure, atrial fibrillation/flutter, in-hospital mortality, Germany, rhythm status, population-based cohort

## Abstract

**Aims:**

Atrial fibrillation or flutter (AFl) frequently coexists with chronic heart failure (CHF), yet its impact on in-hospital outcomes in large, unselected populations remains insufficiently defined.

**Methods:**

We analyzed all hospitalizations for CHF (ICD-10-GM I50*) in Germany from 2014 to 2022 using nationwide administrative data. Patients were stratified by rhythm status (AFl vs. non-AFl). Outcomes included in-hospital mortality, complications, length of stay, and health care costs. Multivariable logistic regression was used to identify independent predictors of mortality.

**Results:**

Among 4,057,291 hospitalizations, 56.9% had AFl. These patients were older (median 82 vs. 79 years), more often female, and showed a higher comorbidity burden, particularly CKD, hypertension, and stroke. AFl was associated with higher rates of complications such as AKI (11.9% vs. 9.7%; p < 0.001) and cardiogenic shock (1.15% vs. 1.03%; p < 0.001), but lower use of mechanical ventilation and assist devices. Despite this, in-hospital mortality was slightly lower in AFl (8.30% vs. 8.66%). AFl patients had longer median hospital stays (8 vs. 7 days), while costs were comparable. Ventilated AFl patients had longer ventilation times (28 vs. 21 hours). In multivariable analysis, AFl was independently associated with higher mortality in most age groups, except for patients aged 40-49, 80-89, and > 90 years.

**Conclusions:**

In this nationwide cohort, AFl was common among CHF patients and linked to more advanced disease. Its impact on in-hospital mortality was age-dependent and complex, highlighting the need for tailored clinical management.

## Introduction

Chronic heart failure (CHF) and atrial fibrillation/flutter (AFl) are among the most prevalent and clinically significant cardiovascular diseases worldwide 1-2. Their growing incidence and prevalence reflect not only an ageing population but also the persistence of shared risk factors such as arterial hypertension (AHT), diabetes mellitus (DM), and structural heart disease. Beyond their individual burden, both conditions are driving forces of health care utilization, characterized by frequent hospitalizations, recurrent outpatient consultations, and substantial socioeconomic costs 1,3.

The clinical impact of CHF and AFl extends beyond symptom burden and health care use. Each condition is independently associated with adverse prognosis, and their coexistence - found in up to 50% of heart failure (HF) patients 4 - is increasingly recognized as a distinct clinical entity with complex management challenges 5. A bidirectional relationship has been established: AFl can lead to CHF through mechanisms such as tachycardia-induced cardiomyopathy, while structural and functional cardiac remodeling in CHF increases susceptibility to atrial arrhythmias 6. Notably, this interplay is not confined to one specific CHF phenotype but affects patients across the spectrum of left ventricular (LV) ejection fraction (EF) 7.

Although prior studies have demonstrated that both optimal guideline-directed HF therapy and rhythm control strategies for AFl can improve clinical outcomes 6, the precise effect of atrial arrhythmia on in-hospital outcomes in patients hospitalized for CHF remains incompletely characterized; particularly in unselected, real-world populations at the national level.

The present study aimed to address this knowledge gap by systematically evaluating the association between AFl and key in-hospital outcomes among patients admitted for CHF. Drawing on a large, unselected cohort derived from nationwide inpatient data in Germany, we sought to generate robust, population-level insights into the clinical relevance of rhythm status in this high-risk group.

## Methods

### Data Source and Study Population

This retrospective, population-based cohort study aimed to characterize the clinical profiles, therapeutic interventions, complications, and in-hospital outcomes of patients hospitalized for CHF, with a specific focus on differences according to rhythm status - namely, the presence or absence of AFl. To this end, we analyzed nationwide administrative hospital data encompassing all admissions in Germany between 1 January 2014 and 31 December 2022 (Figure: graphical abstract).

The study was based on the German inpatient hospital discharge dataset, compiled as part of the national hospital reimbursement system. Since 2002, all hospitals in Germany are legally required to submit standardized information on each inpatient stay to the Institute for the Hospital Remuneration System (Institut für das Entgeltsystem im Krankenhaus, InEK; Siegburg, Germany; http://www.g-drg.de). These data are made accessible for scientific research through the Research Data Centre (RDC) of the Federal Statistical Office and the Statistical Offices of the Federal States (DESTATIS; https://www.destatis.de; DOI: 10.21242/23141.2014.00.00.6.1.0 to 10.21242/23141.2022.00.00.6.1.0).

The dataset included all inpatient cases from hospital admissions, excluding admissions to psychiatric and psychosomatic units. Analyses were performed at the level of hospitalizations, as the dataset does not include unique patient identifiers. A total of 4,057,291 hospitalizations with a primary diagnosis of CHF - defined using the International Classification of Diseases, 10th Revision, German Modification (ICD-10-GM) codes I50.* - were identified. Patients were stratified by the presence (AFl) (ICD-10-GM code I48.*) or the absence of AFl (non-AFl). Diagnoses and procedures were identified using the ICD-10-GM, and the German procedure classification (Operationen- und Prozedurenschlüssel, OPS; Supplementary Tables S1 and S2). If a code was not recorded during the hospital stay, the corresponding variable was treated as absent.

Analytic scripts were developed and validated using a synthetic dataset structurally equivalent to the original DESTATIS data. As direct access to the raw data was not permitted, all statistical analyses were performed by DESTATIS staff based on our pre-specified scripts. Additional methodological details are available in previous work by our group 8-10.

Baseline characteristics included cardiovascular risk factors (AHT, DM, dyslipidemia, obesity, and nicotine use) and cardiovascular comorbidities (coronary heart disease (CHD), peripheral artery disease (PAD), chronic kidney disease (CKD), ischaemic and haemorrhagic stroke, secondary diagnosis of chronic right and left heart failure (CL/RHF), previous stroke, previous coronary artery bypass grafting (CABG), cerebrovascular disease (CeVD), previous valve replacement, myocardial infarction (MI), and previous percutaneous coronary intervention (PCI)) (Table 1).

**Figure 1:**
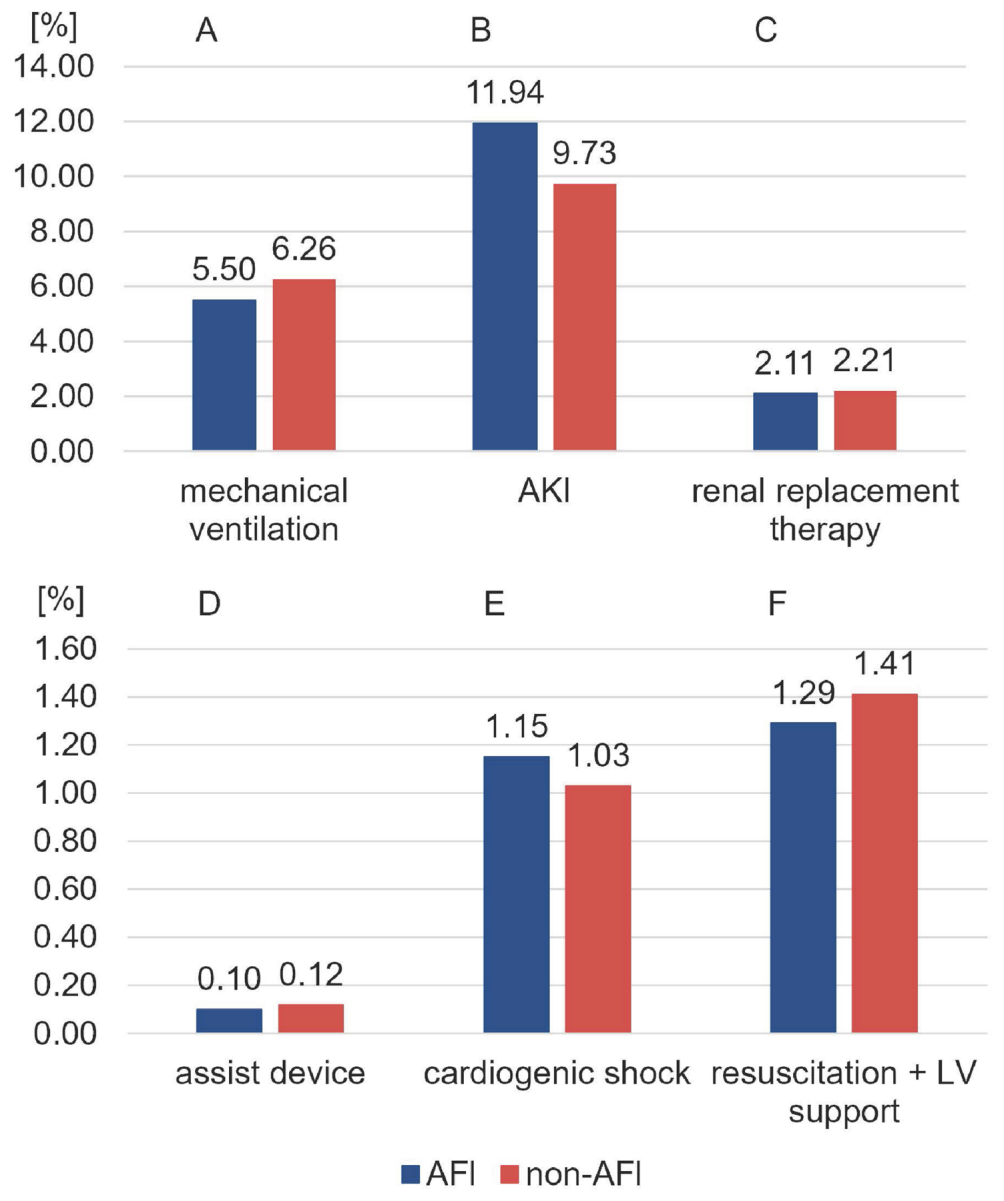
** Occurrence of selected complications among patients hospitalized for CHF, stratified by rhythm status.** Bar charts compare relative frequencies of mechanical ventilation, AKI, and renal replacement therapy (top panel), as well as assist device, cardiogenic shock and resuscitation with LV support (bottom panel) between patients with AFl (blue) and without AFl (non-AFl; red). AFl: Atrial flutter/fibrillation, AKI: acute kidney injury, CHF: chronic heart failure, LV: left ventricular.

We also defined procedures indicative of cardiovascular care intensity; left-heart catheterization (LHC), PCI, implantation of drug-eluting or bare-metal stents (DES, BMS), use of drug-eluting balloons (DEB), plain old balloon angioplasty (POBA), and CABG (Supplementary Table S3). Hospital-recorded complications included cardiogenic shock, acute kidney injury (AKI), renal replacement therapy, resuscitation with LV support, mechanical circulatory support (e.g. extracorporeal membrane oxygenation (ECMO), IMPELLA, intra-aortic balloon pump (IABP)), and mechanical ventilation (Figure 1; Supplementary Table S4). Outcomes of interest included the implantation of univentricular or biventricular intracorporeal pumps, total artificial heart replacement, orthotopic or heterotopic heart transplantation (HTx), heart-lung transplantation, and in-hospital mortality (Table 2).

Median values for age, length of in-hospital stay, and health care costs were compared between AFl and non-AFl. Cost data were based on the total reimbursements received by hospitals per case from statutory health insurance funds, reflecting the scope of services provided (Supplementary Table S5).

### Ethics and Consent

This study was based exclusively on anonymized secondary data from the nationwide German hospital discharge database provided by the Research Data Centre of the Federal Statistical Office (DESTATIS). In accordance with German data protection legislation and institutional regulations, no direct patient contact occurred, and no identifiable individual-level data were accessible to the investigators. Therefore, informed consent was waived, and *a priori* approval by an institutional ethics committee was not required. The study was conducted in accordance with the ethical principles of the *Declaration of Helsinki* (Br Med J 1964; ii:177).

Individual-level raw data could not be accessed or exported. For all figures, the underlying aggregated numerical values used for statistical analyses and visualizations are provided in the Online Supplement, thereby ensuring transparency and reproducibility within the permitted data protection framework.

Due to the use of nationwide administrative hospital data, residual confounding and coding-related bias cannot be excluded. Moreover, analyses were performed at the level of hospitalizations rather than individual patients, as unique patient identifiers were not available, precluding longitudinal linkage of repeated admissions.

### Statistical Analysis

No prospective sample size or power calculation was performed, as this study represents a nationwide census of all hospitalizations fulfilling the predefined inclusion criteria within the study period. Consequently, the sample size was determined by data availability rather than by *a priori* assumptions, rendering formal sample size calculations not applicable.

To assess differences between AFl and non-AFl, categorical variables were analyzed using the Chi-square test, while continuous variables were evaluated using the Wilcoxon rank-sum test.

**Figure 2:**
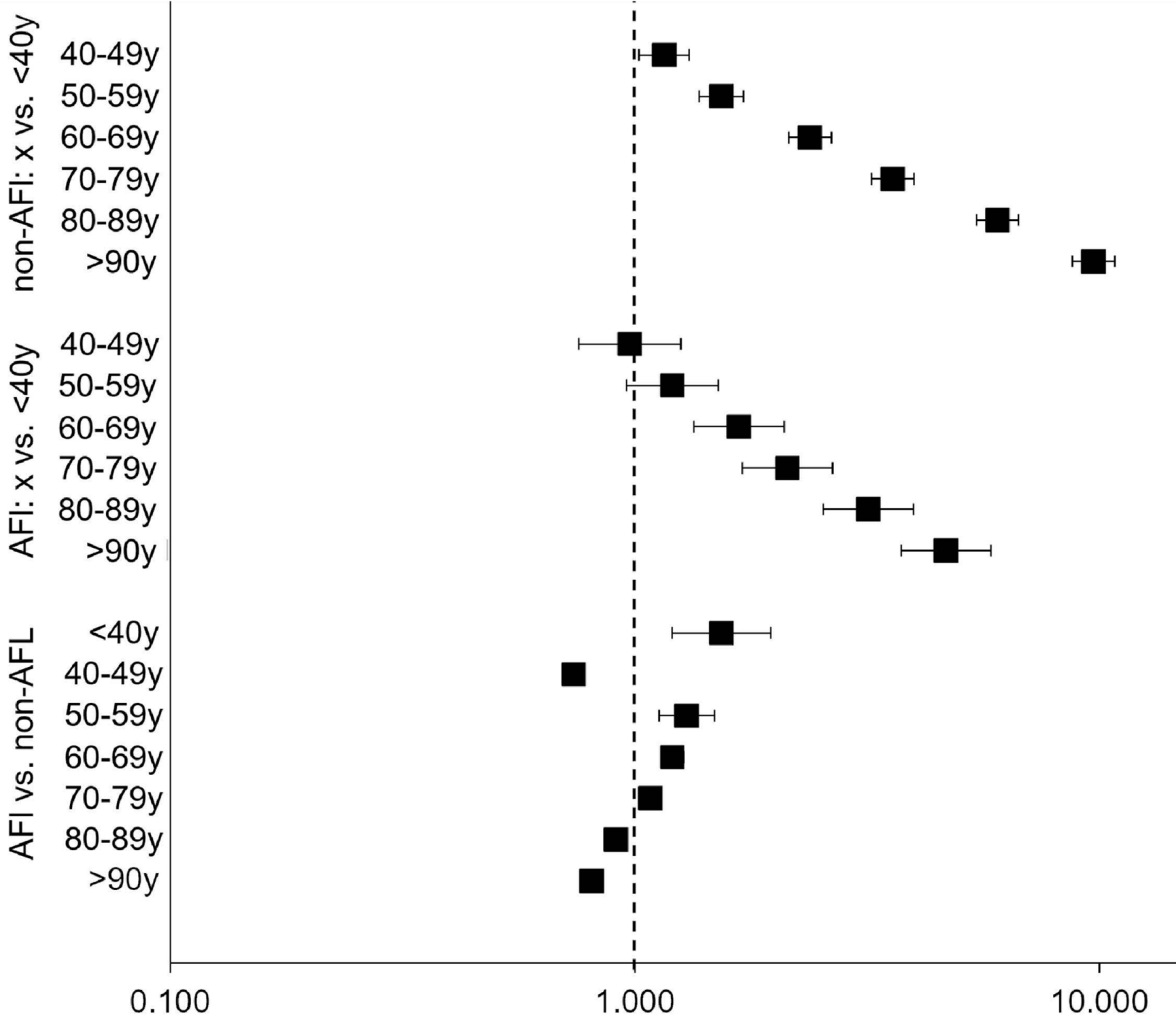
** Age-dependent association between AFl status and in-hospital mortality among patients hospitalized with CHF.** Forest plot displaying odds ratios (OR) for in-hospital mortality stratified by age and rhythm status. The top and middle panels show the effect of increasing age (reference: <40 years) on in-hospital mortality in non-AFl and AFl patients, respectively. The bottom panel compares the odds of death in AFl versus non-AFl patients within each age stratum. Squares indicate point estimates; horizontal lines represent 95% confidence intervals (Supplementary Table S6). AFl: atrial fibrillation/flutter, CHF: chronic heart failure.

**Figure 3:**
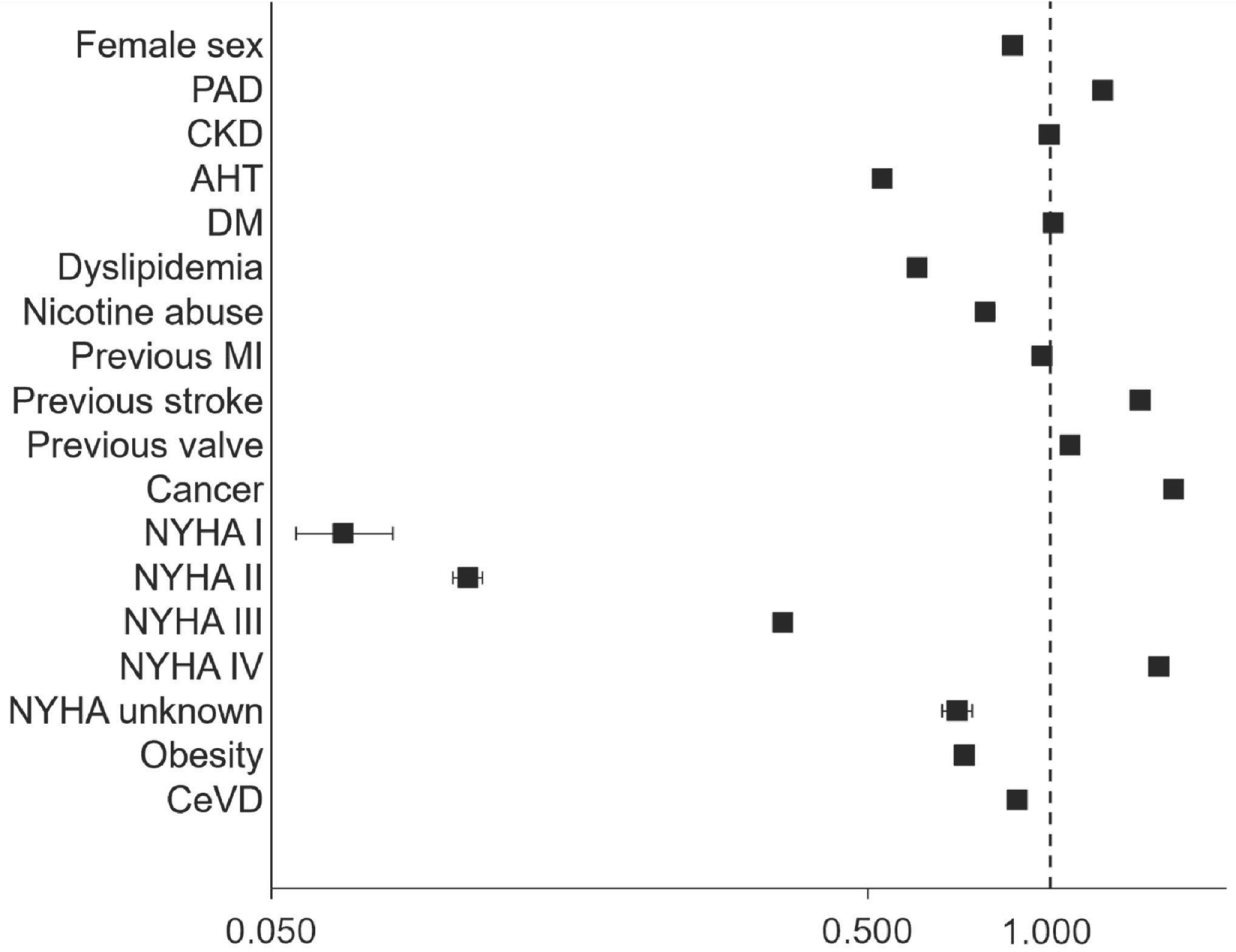
** Multivariable predictors of in-hospital mortality among patients hospitalized with CHF.** Adjusted odds ratios with 95% confidence intervals are shown for demographic variables, cardiovascular risk factors, comorbidities, and NYHA functional class. The reference category for each binary variable is not shown but includes, for example, male sex, no comorbidity, and “no CLHF” for NYHA comparisons. Variables with ORs > 1 were associated with increased in-hospital mortality; those with ORs < 1 were associated with reduced risk. Squares indicate point estimates; horizontal lines represent 95% confidence intervals (Supplementary Table S7). AHT: Arterial hypertension, CHF: chronic heart failure, CKD: chronic kidney disease, CeVD: cerebrovascular disease, DM: diabetes mellitus, PAD: peripheral artery disease, MI: myocardial infarction, NYHA: New York Heart Association.

Multivariable logistic regression models were used to assess predictors of in-hospital mortality in patients hospitalized with CHF. Separate models were constructed to estimate age-stratified mortality risk in patients with and without AFl, as well as to compare mortality between rhythm groups within each age stratum (Figure 2). A second model included cardiovascular risk factors, comorbidities, and NYHA class to identify independent predictors of in-hospital mortality (Figure 3). Odds ratios and 95% confidence intervals were calculated. The models adjusted for all included covariates simultaneously, with appropriate reference categories (e.g. male sex, absence of comorbidity, NYHA “no CLHF”). Given the exploratory nature of this secondary analysis of administrative data, p-values were interpreted descriptively and not as formal hypothesis testing. This approach aligns with STROBE recommendations for observational studies to avoid overinterpretation of statistical significance.

P-values were two-sided, and values below 0.05 were considered potentially statistically noticeable. Statistical computations were performed using SAS (version 9.3; SAS Institute Inc., Cary, NC, USA) and R statistical software (version 4.3.2; released 31 October 2023).

## Results

### Study Population and Baseline Characteristics

Our study included a total of 4,057,291 hospitalizations with a primary diagnosis of CHF (I50.*); 2,307,803 (56.88%) had a diagnosis of AFl. Patients with AFl had a higher median age (82 vs. 79 years) and were more likely to be female (51.40% vs. 49.11%; p < 0.001) (Table 1).

AFl was associated with a higher burden of comorbidities, including CKD (54.10% vs. 44.48%; p < 0.001), AHT (76.72% vs. 73.52%; p < 0.001), CRHF (29.03% vs. 23.83%; p<0.001), and CLHF (25.26% vs. 19.81%; p < 0.001) (Table 1). Furthermore, ischemic and hemorrhagic strokes, prior valve interventions, and previous PCIs or CABG were more prevalent in the AFl group. In contrast, non-AFl patients more frequently had nicotine abuse, DM, CHD, obesity, and a history of previous MI (Table 1).

The distribution across NYHA classes differed between groups, with patients without AFl more frequently classified as NYHA I-III, whereas NYHA IV prevalence was comparable between groups (33.64% vs. 33.70%; p = 0.271) (Table 1).

Non-AFl were more likely to receive invasive cardiac procedures during hospitalization, including LHC, PCI, DES and POBA (Supplementary Table S3). CABG rates were low and comparable between groups (Supplementary Table S3).

### In-hospital Outcomes and Complications

AFl was associated with a higher incidence of AKI (11.9% vs. 9.7%; p < 0.001) and cardiogenic shock (1.15% vs. 1.03%; p < 0.001) (Figure 1; Supplementary Table S4). Paradoxically, patients with AFl had lower rates of renal replacement therapy (2.11% vs. 2.21%; p < 0.001) (Figure 1; Supplementary Table S4). They further had lower rates of mechanical ventilation, implantation of assist devices and combined resuscitation with LV support (Figure 1; Supplementary Table S4).

Despite a higher comorbidity burden and age, in-hospital mortality was slightly lower in the AFl group (8.30% vs. 8.66%; p < 0.001) (Table 2). The use of assist devices and organ transplant procedures was rare in both groups, reflecting the advanced age and clinical frailty of the population (Table 2).

Patients with AFl had a longer median hospital stay (8.00 [6.00-13.00] vs. 7.00 [4.00-11.00] days; Supplementary Table S5), potentially due to increased complication rates and comorbidity burden. Median health care costs were comparable between both groups (2,928.96 [2,497.79-3,386.37] € vs. 2,922.67 [2,498.48-3,421.30] €; Supplementary Table S5).

Among CHF patients who underwent mechanical ventilation, the median duration of ventilation was longer in those with AFl compared to those without (28.00 [7.00-91.00] vs. 21.00 [5.00-71.00] hours).

### Multivariable Analysis of In-hospital Mortality

In multivariable logistic regression, both age and the presence of AFl were independently associated with higher in-hospital mortality in most age groups (Figure 2, Supplementary Table S6). Among patients with AFl, the odds of death increased steeply across age groups, reaching a maximum in those aged > 90 years.

AFl was associated with lower in-hospital mortality compared to non-AFl in specific age groups, namely 40-49, 80-89, and > 90 years. In all other age strata, the presence of AFl conferred a neutral or slightly elevated mortality risk (Figure 2, Supplementary Table S6).

Several comorbid conditions were independently associated with increased in-hospital mortality, including PAD (OR: 1.24; 95% CI: 1.22-1.27), CKD (OR: 1.10; 95% CI: 1.00-1.20), prior stroke (OR: 1.43; 95% CI: 1.40-1.47), previous valve surgery (OR: 1.31; 95% CI: 1.28-1.35), and cancer (OR: 1.38; 95% CI: 1.34-1.42), all with p-values < 0.0001 (Figure 3, Supplementary Table S7). In contrast, AHT (OR: 0.53; 95% CI: 0.53-0.54), dyslipidemia (OR: 0.60; 95% CI: 0.59-0.60), and nicotine abuse (OR: 0.79; 95% CI: 0.76-0.82) were associated with reduced odds of in-hospital mortality (Figure 3, Supplementary Table S7). Female patients had a lower mortality than males (OR: 0.87; 95% CI: 0.87-0.88; p < 0.0001) (Figure 3, Supplementary Table S7).

NYHA class was strongly predictive of mortality. Patients classified as NYHA class IV had the highest risk (OR: 1.54; 95% CI: 1.52-1.56), whereas those in NYHA I or II had significantly lower odds of death, reflecting less advanced disease (Figure 3, Supplementary Table S7).

## Discussion

In this large nationwide cohort of more than four million hospitalizations for CHF, over half of patients were diagnosed with AFl. These patients were older, more often female, and exhibited a markedly higher burden of comorbidities, including CKD, AHT and stroke. Our findings emphasize that AFl in CHF is not merely coincidental arrhythmia but rather a marker of advanced cardiac disease and clinical frailty. We focused on in-hospital outcomes, as nationwide longitudinal follow-up data are not available in Germany, and short-term outcomes remain highly relevant for acute clinical decision making and resource allocation.

Despite their higher age and comorbidity burden, patients with AFl showed slightly lower in-hospital mortality. This seemingly paradoxical finding may reflect earlier presentation, closer monitoring, or differences in clinical management. Importantly, the association between AFl and mortality was age-dependent: AFl was linked to lower mortality in patients aged 40-49, 80-89, and > 90 years, while it conferred neutral or modestly increased risk in other age groups. These findings highlight that the prognostic impact of atrial arrhythmia in CHF is heterogeneous and modulated by age.

Differences in comorbidity profiles between rhythm groups suggest distinct underlying CHF phenotypes. While most previous studies have addressed atrial flutter or atrial fibrillation in highly selected HF populations, large-scale data on the combined burden of atrial fibrillation and flutter remain scarce 11. In this study, we used “AFl” to collectively refer to atrial fibrillation and/or atrial flutter arrhythmias, reflecting their frequent coexistence and overlapping clinical context in hospitalized CHF patients. The AFl group was enriched for conditions such as AHT and female sex - characteristics typically associated with HF with preserved EF (HFpEF) - while the non-AFl cohort more commonly exhibited nicotine abuse and prior MI, features consistent with a more ischemic phenotype. Unfortunately, due to data limitations, EF could not be assessed.

The observed differences in comorbidity profiles between AFl and non-AFl patients raise the possibility that distinct therapeutic responses may exist across HF phenotypes.

Emerging evidence indicates that contemporary HF therapies may influence outcomes irrespective of rhythm status 12-15. Subgroup analyses from large, randomized trials have demonstrated consistent benefits of sodium-glucose cotransporter 2 (SGLT2) inhibitors in patients with and without atrial arrhythmia, while early data suggest potential rhythm-modifying effects of glucagon-like peptide-1 (GLP-1) receptor agonists 13-15. In addition, guideline-recommended CHF therapies such as beta-blockers overlap substantially with pharmacological rate control strategies for AFl, underscoring the close pathophysiological and therapeutic interplay between both conditions 16.

In our cohort, patients with AFl underwent fewer invasive cardiac procedures but experienced longer hospital stays despite comparable costs, suggesting a more prolonged yet less intervention-intensive in-hospital course. Whether this reflects appropriate treatment individualization in older or frailer patients or an underuse of evidence-based interventions remains unclear. Randomized trials such as CASTLE-AF have demonstrated improved outcomes with catheter ablation in selected CHF patients, highlighting the importance of individualized rhythm management strategies 17. Although EF and ablation status were not available in our dataset, the observed variation in mortality by rhythm status and age may partly reflect underlying differences in rhythm management strategies. In younger patients, for example, the lower in-hospital mortality associated with AFl could suggest earlier or more targeted rhythm control, aligning with the paradigm supported by CASTLE-AF 17. While this may represent appropriate tailoring of care in frailer individuals, it could also indicate underutilization of evidence-based interventions in selected candidates.

In multivariable modelling, classical predictors of mortality - age, NYHA class, CKD, PAD, stroke, and cancer - remained prominent. Yet, AHT, dyslipidemia, and nicotine abuse were inversely associated with mortality, possibly reflecting survivorship bias or unmeasured confounding. Our findings raise the hypothesis that the prognostic impact of AFl is not uniform but dynamically modulated by age and comorbidity burden. Beyond rhythm status alone, integrated clinical risk assessment may further improve prognostic stratification in CHF populations. In this context, the CHA₂DS₂-VASc score - originally developed for thromboembolic risk estimation in AFl - has also been associated with mortality and adverse cardiovascular outcomes in CHF patients irrespective of atrial arrhythmia status. Recent studies demonstrated that increasing CHA₂DS₂-VASc scores correlate with higher mortality and rehospitalization rates even in CHF patients without AFl, likely reflecting the cumulative impact of age, vascular disease, and comorbidity burden 18,19. This concept is consistent with our findings, in which age, NYHA class, CKD, PAD, stroke, and other comorbidities appeared as important determinants of in-hospital mortality independent of rhythm status, further stressing the importance of comprehensive risk assessment beyond the presence or absence of atrial arrhythmia alone. The paradoxical survival advantage seen in the youngest and oldest strata may stem from more regular follow-up, selective survival, or other unmeasured protective factors.

### Limitations

This study has several limitations that warrant consideration. First, it is based on administrative data from a national inpatient registry, which - although comprehensive in scope - lacks clinical granularity. Parameters such as left ventricular ejection fraction, biomarker levels, medical therapy, rhythm type and duration (paroxysmal, persistent, or permanent), and ablation status were not available, limiting our ability to stratify patients by CHF phenotype, arrhythmia characteristics, or therapeutic intensity. Second, the analysis was performed at the level of hospitalizations rather than unique patients, as the dataset does not contain individual patient identifiers. Consequently, multiple admissions of the same patient could not be linked, potentially leading to overrepresentation of frequently hospitalized individuals, although this approach reflects the real-world burden on the health care system. Third, the observational design precludes causal inference; residual confounding from unmeasured factors such as frailty, socioeconomic status, or physician decision-making is likely, and treatment allocation during hospitalization may have been influenced by these factors. The lack of information on antiarrhythmic or other cardiovascular medications further limits adjustment for treatment effects. Fourth, atrial fibrillation and atrial flutter were combined under the term “AFl” because the ICD-10-GM coding in the nationwide dataset does not reliably distinguish between these arrhythmias. While they frequently coexist and share overlapping risk factors and management strategies in CHF, they may differ in pathophysiology, prognosis, and optimal treatment - representing a major limitation of this study. Fifth, the descriptive interpretation of p-values reflects the exploratory nature of the analysis and aims to emphasize effect sizes and clinical relevance rather than formal statistical significance. Finally, only in-hospital outcomes were assessed; long-term endpoints such as post-discharge mortality, rehospitalization, or quality of life could not be evaluated, precluding conclusions on the sustained impact of rhythm status beyond the index hospitalization.

## Supplementary Material

Supplementary tables.

## Declaration of Generative AI and AI-assisted Technologies in the Manuscript Preparation Process

During the preparation of this manuscript, the authors used ChatGPT (OpenAI) to support language editing and improve clarity and structure of the text. All content was critically reviewed and edited by the authors, who take full responsibility for the final version of the manuscript.

## Figures and Tables

**Table 1:** Cardiovascular risk profile and comorbidities in CHF hospitalizations, stratified by AFl status.

	AFl	non-AFl	p-value
	2,307,803	1,749,488	
Sex ♂	48.60 (1,121,703)	50.89 (890,300)	< 0.0001
Cardiovascular risk factors			
DM % (N)	38.11 (879,480)	38.43 (672,414)	< 0.0001
dyslipidemia % (N)	28.32 (653,628)	28.84 (504,569)	< 0.0001
obesity % (N)	10.38 (239,524)	11.58 (202,611)	< 0.0001
nicotine abuse % (N)	1.21 (28,029)	2.71 (47,407)	< 0.0001
AHT % (N)	76.72 (1,770,634)	73.52 (1,286,198)	< 0.0001
cardiovascular comorbidities			
ischemic stroke % (N)	0.51 (11,777)	0.40 (7,049)	< 0.0001
PAD % (N)	5.74 (132,542)	5.75 (100,631)	0.7056
hemorrhagic stroke % (N)	0.11 (2,595)	0.08 (1,341)	< 0.0001
CRHF % (N)	29.03 (669,893)	23.83 (416,977)	< 0.0001
CLHF % (N)	25.26 (583,000)	19.81 (346,497)	< 0.0001
NYHA I % (N)	0.25 (5,701)	1.14 (19,997)	< 0.0001
NYHA II % (N)	2.76 (63,795)	5.95 (104,123)	< 0.0001
NYHA III % (N)	25.55 (589,566)	26.71 (467,346)	< 0.0001
NYHA IV % (N)	33.64 (776,430)	33.70 (589,504)	0.271
NYHA unknown % (N)	0.35 (8,053)	0.68 (11,861)	< 0.0001
CKD % (N)	54.10 (1,248,610)	44.48 (778,112)	< 0.0001
CHD % (N)	30.13 (695,322)	30.71 (537,329)	< 0.0001
previous stroke % (N)	2.98 (68,803)	2.51 (43,958)	< 0.0001
previous CABG % (N)	7.83 (180,749)	7.34 (128,340)	< 0.0001
CeVD % (N)	1.22 (28,118)	1.37 (23,949)	< 0.0001
previous valve % (N)	2.33 (53,803)	1.47 (25,803)	< 0.0001
previous MI % (N)	7.73 (178,379)	8.90 (155,691)	< 0.0001
previous PCI % (N)	20.55 (474,168)	19.98 (244,539)	< 0.0001

Cardiovascular risk factors and cardiovascular comorbidities in patients hospitalized due to CHF analyzed according to the presence or absence of AFl. Absolute and relative frequencies**.** AFl: Atrial flutter/fibrillation, AHT: arterial hypertension, CHF: chronic heart failure, CLHF: chronic left ventricular heart failure, CKD: chronic kidney disease, CRHF: chronic right ventricular heart failure, CeVD: cerebrovascular disease, CHD: coronary heart disease, DM: diabetes mellitus, LV: left ventricular, MI: myocardial infarction, NYHA: New York Heart Association, PAD: peripheral artery disease, PCI: percutaneous coronary intervention.

**Table 2:** Outcomes in CHF hospitalizations, stratified by AFl status.

	AFl	non-AFl	p-value
univentricular intracorporeal pump % (N)	0.08 (1,789)	0.06 (1,027)	< 0.0001
biventricular intracorporeal pump % (N)	< 0.01 (≤ 2)	< 0.01 (≤ 2)	-
total artificial heart % (N)	< 0.01 (11)	< 0.01 (14)	0.1935
orthotopic HTx % (N)	0.01 (280)	0.01 (230)	0.3669
heterotopic HTx % (N)	0.00 (0)	0.00 (0)	-
heart-lung-Tx % (N)	< 0.01 (≤ 2)	< 0.01 (≤ 2)	-
in-hospital mortality % (N)	8.30 (191,541)	8.66 (151,553)	< 0.0001

Outcomes in patients hospitalized due to CHF analyzed according to the presence or absence of AFl. Absolute and relative frequencies**.** AFl: Atrial flutter/fibrillation, CHF: chronic heart failure, HTx: heart transplantation, Tx: transplantation.

## Data Availability

The data underlying this article are available from the Research Data Centre of the Federal Statistical Office (DESTATIS, Germany) under strict data protection conditions and cannot be shared publicly.
