# Supplementary material for: Interplay of Atrial Arrhythmia and Chronic Heart Failure: A Population-Based Analysis of Hospital Outcomes in Germany
Source: Int J Med Sci. 2026 Jun 25;23(8):2540–7. doi: 10.7150/ijms.131856 (PMC13411478; doi:10.7150/ijms.131856)
Supplement: Supplementary file 1 — Supplementary tables. [file ijmsv23p2540s1.pdf]

Table S1. ICD-10-GM Codes used to define health conditions.

Health Problems 10th Revision (ICD-10 GM)	Code
Arterial hypertension (AHT)	I10.-, I11.-, I12.-, I13.-, I15.-
Diabetes mellitus (DM)	E10.-, E11.-,
Dyslipidemia	E78.-
Nicotine abuse	F17.-
Myocardial Infarction (MI)	I25.2, I24.1
Obesity	E66.-
Chronic right heart failure (CRHF)	I50.0-
Chronic left heart failure (CLHF)	I50.11-I50.14
Chronic heart failure (CHF)	I50.-
Previous stroke	I69.3, I69.4
Coronary heart disease (CHD)	I25.-
Atrial flutter/fibrillation (AFl)	I48.-
Peripheral artery disease (PAD)	I70.2-
Cerebrovascular disease (CeVD)	I65.-,
Chronic kidney disease (CKD)	N18.-, N19.-
Acute kidney injury (AKI)	N17
Cardiogenic shock	R57.0, T81.1

Table S2. OPS codes used to identify medical procedures (German Procedure Classification System).

German procedure classification system (OPS)	Code
Percutaneous coronary intervention (PCI)	8-837
Left-heart-Catheterization (LCH)	1-275
Biventricular intracorporal pump	5-376.50
Univentricular intracorporal pump	5-376.40
Impella	8-83a.3
Artificial heart	5-376.6
HTx heterotopic	5-375.1
HTx orthotopic	5-375.0
Heart- and Lung Tx	5-375.2
Intra-aortic balloon pump (IABP)	8-83a.0
Renal replacement therapy	8-853, 8-855, 8-854, 8-857
Left ventricle support	8-851, 8-852, 8-83a.0, 8-83a.3
Resuscitation	8-771

Table S3: Recorded interventions in CHF hospitalizations, stratified by AFl status.

	AFl	non-AFl	p-value
**LHC % (N)**	12.18 (280,990)	17.88 (312,741)	<0.0001
**PCI % (N)**	2.79 (64,381)	4.04 (70,620)	<0.0001
**DES % (N)**	2.26 (52,245)	3.51 (61,367)	<0.0001
**BMS % (N)**	0.17 (3,930)	0.15 (2,549)	<0.0001
**DEB % (N)**	0.17 (3,832)	0.27 (4,697)	<0.0001
**POBA % (N)**	2.34 (53,994)	3.56 (62,359)	<0.0001
**CABG % (N)**	0.04 (749)	0.04 (894)	0.0434

Distribution of recorded intervention in patients hospitalized due to CHF analyzed according to the presence or absence of AFl. Absolute and relative frequencies**.** AFl: Atrial flutter/fibrillation, BMS: bare-metal stent, CABG: coronary artery bypass grafting, CHF: chronic heart failure, DES: drug-eluting stent, DEB: drug-eluting balloon, LHC: left-heart catheterization, POBA: plain old balloon angioplasty, PCI: percutaneous coronary intervention.

Table S4: In-hospital Complications in CHF hospitalizations, stratified by AFl status.

	AFl	non-AFl	p-value
**Cardiogenic shock % (N)**	1.15 (26,532)	1.03 (18,057)	<0.0001
**AKI % (N)**	11.94 (275,439)	9.73 (170,236)	<0.0001
**Renal replacement therapy % (N)**	2.11 (48,653)	2.21 (38,662)	<0.0001
**Resuscitation + LV support % (N)**	1.29 (29,862)	1.41 (24,661)	<0.0001
**Assist device % (N)**	0.10 (2,207)	0.12 (2,098)	<0.0001
**Mechanical ventilation % (N)**	5.50 (126,818)	6.26 (109,514)	<0.0001

In-hospital complications in patients hospitalized due to CHF analyzed according to the presence or absence of AFl. Absolute and relative frequencies**.** AFl: Atrial flutter/fibrillation, AKI: Acute kidney injury. CHF: chronic heart failure, LV: left ventricular.

Table S5: Health care costs, length of hospitalization, and age in CHF patients stratified by presence or absence of AFl.

	Costs [euro]	length of hospitalization [days]	Duration of mechanical ventilation [hours]	age [years]
**AFl**	2928.96 [2497.79-3386.37]	8.00 [6.00-13.00]	28.00 [7.00-91.00]	82.00 [76.00-87.00]
**non-AFl**	2922.67 [2498.48-3421.30]	7.00 [4.00-11.00]	21.00 [5.00-71.00]	79.00 [69.00-85.00]

Health care costs (in Euro), length of hospital stay for CHF (in days), duration of mechanical ventilation (in hours), and patient age (in years) were analyzed according to the presence or absence of AFl. Values are reported as medians with IQR. AFl: Atrial flutter/fibrillation, CHF: chronic heart failure, IQR: interquartile ranges.

Table S6: Adjusted ORs for in-hospital mortality in patients hospitalized with CHF, stratified by age group and rhythm status.

		OR	95%-CI	p-value
nonAFl: x vs. <40y	40-49y	1.158	1.022; 1.312	0.0213
	50-59y	1.536	1.378; 1.713	<0.0001
	60-69y	2.384	2.145; 2.648	<0.0001
	70-79y	3.595	3.239; 3.990	<0.0001
	80-89y	6.042	5.445; 6.703	<0.0001
	>90y	9.720	8.757; 10.789	<0.0001
AFl: x vs. <40y	40-49y	0.976	0.758; 1.256	0.8498
	50-59y	1.205	0.960; 1.513	0.1074
	60-69y	1.677	1.341; 2.098	<0.0001
	70-79y	2.132	1.706; 2.665	<0.0001
	80-89y	3.182	2.546; 3.977	<0.0001
	>90y	4.676	3.740; 5.845	<0.0001
AFl vs. non-AFl	<40y	1.536	1.202; 1.964	0.0006
	40-49y	0.739	0.727; 0.751	<0.0001
	50-59y	1.295	1.129; 1.485	0.0002
	60-69y	1.206	1.139; 1.276	<0.0001
	70-79y	1.081	1.051; 1.112	<0.0001
	80-89y	0.911	0.897; 0.926	<0.0001
	>90y	0.809	0.801; 0.818	<0.0001

The first two sections show age-stratified mortality risk in non-AFl and AFl patients, respectively, with patients aged <40 years serving as the reference group. The third section compares mortality between AFl and non-AFl patients within each age group. ORs were

derived from multivariable logistic regression models adjusted for relevant covariates. Values are presented with 95% confidence intervals (CIs) and corresponding p-values based on Wald tests. AFl: Atrial flutter/fibrillation, CI: confidence interval, OR: odds ratio.

Table S7: Adjusted ORs for in-hospital mortality in patients hospitalized with CHF, based on multivariable logistic regression.

	OR	95%-CI	p-value
**Female Sex**	0.874	0.867; 0.880	<0.0001
**PAD**	1.240	1.221; 1.258	<0.0001
**CKD**	1.008	1.000; 1.015	0.0477
**AHT**	0.529	0.525; 0.533	<0.0001
**DM**	1.025	1.017; 1.033	<0.0001
**Dyslipidemia**	0.605	0.600; 0.611	<0.0001
**Nicotine abuse**	0.787	0.758; 0.817	<0.0001
**Previous MI**	0.981	0.968; 0.995	0.0081
**Previous stroke**	1.433	1.406; 1.460	<0.0001
**Previous valve**	1.092	1.064; 1.121	<0.0001
**Cancer**	1.631	1.604; 1.658	<0.0001
**NYHA I**	0.066	0.055; 0.080	<0.0001
**NYHA II**	0.107	0.101; 0.113	<0.0001
**NYHA III**	0.360	0.355; 0.364	<0.0001
**NYHA IV**	1.541	1.529; 1.553	<0.0001
**NYHA unknown**	0.706	0.666; 0.749	<0.0001
**Obesity**	0.727	0.716; 0.738	<0.0001
**CeVD**	0.891	0.861; 0.922	<0.0001

Variables included selected cardiovascular risk factors, comorbidities, and NYHA functional class. “No CLHF” served as the reference category for NYHA comparisons. Values are reported with 95% confidence intervals (CIs) and corresponding p-values derived from Wald tests. ORs >1 indicate increased mortality risk; ORs <1 indicate protective associations. AHT: Arterial hypertension, AFl: Atrial flutter/fibrillation, CHF: chronic heart failure, CLHF: chronic left ventricular heart failure, CI: confidence interval, CKD: chronic kidney disease, CeVD: cerebrovascular disease, DM: diabetes mellitus, OR: odds ratio, PAD: peripheral artery disease, MI: myocardial infarction, NYHA: New York Heart Association.
